# Long-Term Air Pollution Exposure and Ischemic Heart Disease Mortality Among Elderly in High Aging Asian Economies

**DOI:** 10.3389/fpubh.2021.819123

**Published:** 2022-02-07

**Authors:** Ayesha Mumtaz, Nadia Rehman, Aftab Haider, Shazia Rehman

**Affiliations:** ^1^School of Public Administration, Hangzhou Normal University, Hangzhou, China; ^2^College of Public Administration, Zhejiang University, Hangzhou, China; ^3^Department of Mathematics, COMSATS University, Islamabad, Pakistan; ^4^Business Studies Department, Bahria University, Islamabad, Pakistan; ^5^Department of Biomedical Sciences, Pak-Austria Fachhochschule, Institute of Applied Sciences and Technology, Haripur, Pakistan

**Keywords:** air pollutants, PM_2.5_, ground-level ozone, ischemic heart disease, grey modeling, G-TOPSIS, elderly, Asia

## Abstract

In the epidemiological literature, the impact of environmental pollution on cardiac mortality has been well documented. There is, however, a paucity of evidence on the impact of air pollution exposure on ischemic heart disease (IHD) mortality among the Asian aged population. In response, this research seeks to investigate the degree of proximity between exposure to ambient PM_2.5_, household PM_2.5_, ground-level ozone (O_3_), and IHD mortality in the top seven Asian economies with the highest aging rates. This investigation is held in two phases. In the first phase, grey modeling is employed to assess the degree of proximity among the selected variables, and then rank them based on their estimated grey weights. In addition, a grey-based Technique for Order of Preference by Similarity to Ideal Solution (G-TOPSIS) is adopted to identify the key influencing factor that intensifies IHD mortality across the selected Asian economies. According to the estimated results, South Korea was the most afflicted nation in terms of IHD mortality owing to ambient PM_2.5_ and ground-level O_3_ exposure, whereas among the studied nations India was the biggest contributor to raising IHD mortality due to household PM_2.5_ exposure. Further, the outcomes of G-TOPSIS highlighted that exposure to household PM_2.5_ is a key influencing risk factor for increased IHD mortality in these regions, outweighing all other air pollutants. In conclusion, this grey assessment may enable policymakers to target more vulnerable individuals based on scientific facts and promote regional environmental justice. Stronger emission regulations will also be required to mitigate the adverse health outcomes associated with air pollution exposure, particularly in regions with a higher elderly population.

## Introduction

Environmental pollution is evolving as one of the most critical environmental challenges of the 21st century, and its consequences are becoming more visible with time. During the last several decades, it has had a significant impact on human health and longevity, and it is now considered among the most major environmental threats to public health (1). More than 5.5 million people expire prematurely each year as a consequence of diseases induced by inhaling contaminated air. It kills nearly six times as many people as malaria and nearly four times as many as HIV (2). Roughly one of every 10 fatalities globally is linked to hazardous air pollutants and mitigating the impact of pollution could save about 13 million lives each year (3). Air pollution is presently attributable to one-third of all fatalities from heart diseases, lung cancer, and chronic respiratory diseases (CRDs). Additionally, the WHO reports that almost 90% of the population in low- and middle-income countries (LMICs) do not have access to clean air (2). The problem appears to be even worse in developing nations, which are home to some of the world's fastest growing metropolitan areas (4, 5).

Asia inhibited 60% of the global population, including several emerging economies. Deaths from ischemic heart diseases (IHDs) are more in Asia than in Western Europe, the USA, and Australia (6). As a result of the rising number of aging populations, Asia is the region particularly affected by the increasing incidence and mortality from cardiovascular diseases (CVDs) (7, 8). It has been confirmed that particulate matter and all air pollutants are causing the risk of increased hospital admissions for IHDs among the elderly. Exposure to ambient air pollutants is considered to be riskier for older people and is more likely to exacerbate cardiac events in older adults than in the young population (9, 10). As a result of rapid industrialization and urbanization, Asian economies have seen an increase in their elderly populations, as well as changes in lifestyle and diet habits (11–14). Consequently, cardiac risk factors are growing among Asian people, making them more susceptible to CVDs. There are ways to minimize the prevalence of this disease by recognizing and improving risk factors in aging societies.

Several epidemiological pieces of research have confirmed a substantial link between acute and chronic effects of air pollutants and CVDs (15–18). In this respect, the WHO and several other public and private organizations have established health-based air quality guidelines for particulate matter and surface O_3_ due to mounting evidence that these pollutants can cause a variety of adverse health outcomes. Technical assessments of the advantages of air quality initiatives or public policies governing pollutant concentrations have become an increasingly essential element of national decision-making strategies. Particulate is a combination of solid and liquid nanoparticles and its chemistry and size can fluctuate; its concentration is a worry in metropolitan areas. Particulate matter is emitted by mobile sources such as vehicles, motorcycles, mini-buses, and trucks, as well as stationary sources such as gas furnaces, power stations, and industries (19, 20). Ground-level ozone (O_**3**_) is one of the most dangerous toxic constituents of photochemical air pollution, and it has been linked to an increase in cardiac and respiratory mortality (21, 22). Despite the fact that air pollution is a primarily urban concern, various worldwide studies have shown evidence that reducing air pollution exposure corresponds to less negative health outcomes (23, 24). This conclusion has compelled policymakers to address the issue of air pollution at a time when the significance of sustainable development and its environmental impact on public health is becoming more widely recognized across the globe.

In view of the importance, a plethora of research and analytic approaches are being used throughout the world to evaluate the association between disease mortality and air pollution exposure. However, there is a scarcity of evidence connecting to air pollution exposure and the risk of IHD mortality in the older population in Asian countries with higher aging rates. The scant evidence has made determining the real situation in these areas extremely challenging. With this in mind, the present research attempts to fill literature gaps by investigating the relationship between exposure to ambient PM_2.5_, ground-level O_**3**_ exposure, household PM_2.5_ exposure, and IHD mortality in the elderly population of the top seven Asian nations (Japan, South Korea, Thailand, China, Sri Lanka, India, and Nepal) with the highest aging rate. However, by considering all of these regions together with a wide variety of air pollutants associated with IHD mortality, we may be able to present a more comprehensive spectrum of the relationships. To investigate this connection, we utilized an advanced mathematical grey relational analysis (GRA) modeling of grey system theory (GST), which included Deng degree of GRA, absolute degree of GRA, and the second synthetic degree of GRA. The GRA models provide many advantages when contrasted with standard statistical models. For instance, they exhibit a greater precision and may yield reliable outcomes even with small sample size. In addition, this study also employed a grey-based Technique for Order of Preference by Similarity to Ideal Solution (G-TOPSIS) to identify the key influencing factor that intensifies cardiac mortality across the selected Asian nations. The proposed methodologies are more appropriate when contrasted with other techniques for convincing outcomes and assisting with avoiding endogeneity issues. The suggested model provides a significant tool and a source of additional pragmatic insights for policymakers and decision makers in drawing rational decisions to reduce air pollution and mortality in these regions.

## Materials and Methods

### Data Source

For the present analysis, the annual time series data for the period of 2010–2019 are utilized. Information on IHD mortality (reference sequence) against each selected Asian country was extracted from the Global Burden of Disease study, harmonized by the Institute for Health Metrics and Evaluation (IHME), and is publicly accessible online (25). In addition, data on the population-weighted exposure (comparative sequence) to ambient PM_2.5_ concentrations (μg/m^3^), ground-level (tropospheric) O_3_ concentrations (ppb) exposure, and household PM_2.5_ concentrations exposure (μg/m^3^) were taken from the State of Global Air report (2010–2019) (26). These parameters are estimated as the national population's average exposure levels (urban and rural regions) to mean annual concentrations of PM_2.5_ and O_3_, separately. To evaluate PM_2.5_ exposures, the GBD scientist combines the number of people living in a specific region and PM_2.5_ concentrations to which they are exposed. O_3_ exposures were evaluated by combining the number of people living in a specific region and the surface O_3_ concentration to which they are exposed. This method determines human exposure to air pollutants in terms of the population-weighted average seasonal 8-h daily maximum concentrations for a specific region. All data analyses are executed in SPSS (v26, IBM, NY, USA), while the graphical representation and study framework are handled in Microsoft Excel (2019) and Microsoft Visio (2019), respectively.

#### Grey Relational Decision Analyses

Grey relational analysis methods are one of the core area subjects of GST, which was introduced by Deng Julong, a Chinese scholar, in 1982 (27) to manage dubious systems with limited input. GST belongs to the category of uncertainty theories, which also incorporates rough set theory, fuzzy theory, interval theory, and other related theories. Additionally, GST, as led by its methodology, addresses the vulnerability in a manner distinct from previous vulnerability speculations. GST categorizes the world's systems into three different groups, which are white, black, and grey. If there is no information provided, it refers to black data. Whereas, if complete information is accessible, it is described as white data or structure. Thusly, a GS turns into a framework that is partially known and rather cryptic (28–30). GST and its related models are well known for their potential to predict and make choices based on smaller sample sizes and poor and inadequate data. The GRA models attempt to grasp unclear correlations between GST features. The general concept behind GRA is the degree of proximity (correlation) of the geometrical framework of the data series suggests that the structure parameters may be used to predict the proximity of a link among the system variables. This proximity is referred to as a correlation in the literature. Deng's GRA (D-GRA), absolute GRA (A-GRA), and second synthetic GRA (SS-GRA) are the three components of the GRA model. In essence, the D-GRA model assesses the effect of one variable reflected by a data set on the other, whereas the A-GRA model evaluates the relationship between the two. Moreover, the SS-GRA model estimates an overall measure of the relationship among the parameters under consideration. A comprehensive review of GRA models can be found in the work of Liu et al. (31). The algorithms involved with the grey methods are explained in the following sections.

##### Deng's GRA Model

Let *Y*_*i*_ = (*y*_*i*(1)_, *y*_*i*(2)_, ⋯ , *y*_*i*(*m*)_) be the basic/reference sequence addressing a dependent variable and *Y*_*j*_ = (*y*_*j*(1)_, *y*_*j*(2)_, ⋯ , *y*_*j*(*m*)_) be the arrangement of comparative sequences addressing independent variables, in the wake of going through initialing an operator, then, at that point grey relational gradient (GRG), the real number degree addressing the output of GRA model is depicted as γ_*ij*_ or γ(*Y*_*i*_, *Y*_*j*_) and can be accompanied by:


$$\begin{array}{l} \gamma \left(Y_{i}\;,\;Y_{j}\right)=\;\frac{1}{m}\overset{m}{\underset{h=1}{\sum }}\gamma \left(y_{i\left(h\right)},y_{j\left(h\right)}\right) \end{array}$$

where


$$\begin{array}{l} \gamma \left(y_{i\left(h\right)},y_{j\left(h\right)}\right)\\ =\;\frac{min_{k}min_{h}|y_{i\left(h\right)}-y_{j\left(h\right)}|+\zeta \;max_{k}max_{h}|y_{i\left(h\right)}-y_{j\left(h\right)}|}{\left|y_{i\left(h\right)}-y_{j\left(h\right)}|+\;\zeta \;max_{k}max_{h}|y_{i\left(h\right)}-y_{j\left(h\right)}\right|}. \end{array}$$

Here, ζ* ϵ* (0, 1) represents a distinguishing coefficient, and its value is generally considered to be ζ = 0.5. The implementation of the D-GRA model for evaluating the effect of one parameter/variable on another has been highlighted in the literature (32–34).

##### A-GRA Model

If *Y*_*i*_ = (*y*_*i*(1)_, *y*_*i*(2)_, ⋯ , *y*_*i*(*m*)_) and *Y*_*j*_ = (*y*_*j*(1)_, *y*_*j*(2)_, ⋯ , *y*_*j*(*m*)_) are the two data sequences representing two variables associated with a system, then the algorithm to calculate the bidirectional absolute grey relational gradient (A-GRG) is listed as follows:


$$\begin{array}{l} \epsilon_{ij}=\frac{1+|r_{i}|+|r_{j}|}{1+|r_{i}|+|r_{j}|+|r_{i}-r_{j}|}, \end{array}$$

where


$$\begin{array}{l} \;r_{i}=\;\underset{1}{\overset{m}{\int }}Y_{i}^{0}dt,\;r_{j}=\underset{1}{\overset{m}{\int }}Y_{j}^{0}dt,\;r_{i}-\;r_{j}\\ \;=\;\underset{1}{\overset{m}{\int }}\left(Y_{i}^{0}-Y_{j}^{0}\right)dt\\ \;Y_{i}^{0}=\left(y_{i\left(1\right)}^{0},y_{i\left(1\right)}^{0}\;,\cdots \;,y_{i\left(m\right)}^{0}\right)\\ \;Y_{j}^{0}=\left(y_{j\left(1\right)}^{0},y_{j\left(1\right)}^{0}\;,\cdots \;,y_{j\left(m\right)}^{0}\right)\\ Y_{i\left(h\right)}^{0}=y_{i\left(h\right)}-\;y_{i\left(1\right)}\text{ and}Y_{i\left(h\right)}^{0}=\;y_{i\left(h\right)}-\;y_{i\left(1\right)}\\ \;h=1,2,\cdots \;,m. \end{array}$$

##### SS-GRA Model

The SS-GRA model is an approach to estimate second synthetic grey relational gradient (SS-GRG) and can be acquired by utilizing the accompanying equation.


$$\begin{array}{l} ℘=\vartheta \epsilon_{ij}+\left(1-\vartheta \right)\gamma_{ij}\;\vartheta \epsilon \left[0,1\right] \end{array}$$

where ℘ stands for the SS-GRA, ″ϵ″ for the A-GRA, and ″γ″ for the D-GRA between the two grey data sets *Y*_*i*_ and *Y*_*j*_. When a decision maker desires a holistic assessment that evenly integrates the benefits of both ″ϵ″and ″γ″ without preferring one over the other and may keep ϑ at 0.5 (35). In the case of preferring is fundamental, then, at that point, the value of ″ϑ″ can be adjusted. In the event that one desires to prefer ″γ″, then ″ϑ″ can be diminished, and assuming one desires to prefer ″ϵ,″ then ″ϑ″ can be increased. In the present investigation, we thought of ϑ = 0.5.

##### Grey Numbers

A grey number represents an interim with unspecified information but a well-defined range of possibilities, which is depicted by a sign ⊗. In the GST, there are multiple forms of grey numbers; however, the present study introduces the following three forms:

*Description 1*: If ⊗*E* represents a grey number whose lower limit can only be evaluated, it is termed as a grey number with a lower limit only and is expressed as ⊗*E* = [*E*, ∞).

*Description 2*: If ⊗*E* is a grey number whose upper limit can only be evaluated, it is termed as a grey number with an upper limit only and is expressed as ⊗*E* = (∞, Ē].

*Description 3*: If ⊗*E* represents a grey number whose lower and upper limits can only be evaluated, it is termed as an interval grey number, which is expressed as ⊗*E* = (*E*, Ē].

Let $⊗\;E=\left[\underline{E},Ē\right]\text{and}⊗H=\left[\underline{H},\bar{H}\right]$ are the two grey numbers, then arithmetic operations ought to be composed in a manner as follows:


$$\begin{array}{l} ⊗E+⊗H=\left[\underline{E}+\underline{H},E-\bar{H}\right]\\ ⊗E-⊗H=⊗E+\left(-⊗H\right)=\left[\underline{E}-\bar{H},Ē-\bar{H}\right]\\ ⊗E\times ⊗H=\left[Min\left\{\underline{EH}Ē\bar{H}Ē\underline{HE}\bar{H}\right\}Max\left\{\underline{EH}Ē\bar{H}Ē\underline{HE}\bar{H}\right\}\right]\\ \;\frac{⊗E}{⊗H}=⊗E\times ⊗H^{-1}=\left[Min\left\{\frac{\underline{E}}{\underline{H}}\frac{\underline{E}}{\bar{H}}\frac{Ē}{\underline{H}}\frac{Ē}{\bar{H}}\right\}\right]\\ \;Max\left\{\frac{\underline{E}}{\underline{H}}\frac{\underline{E}}{\bar{H}}\frac{Ē}{\underline{H}}\frac{Ē}{\bar{H}}\right\} \end{array}$$

The length of the grey number ⊗*E* = [*E*, Ē] is introduced in the following equation:


$$\begin{array}{l} R\left(⊗E\right)=Ē-\underline{E} \end{array}$$

If there are two grey numbers $⊗E=\left[\underline{E},Ē\right]\text{and}⊗H=\left[\underline{H},\bar{H}\right]$, the degree of grey synthetic assessment between these two numbers can be estimated by utilizing the following expression:


$$\begin{array}{l} P\left\{⊗E\leq ⊗H\right\}=\frac{\text{Max}\left\{0,R^{*}-\text{Max}\left(0,Ē,\underline{H}\right)\right\}}{R^{*}},\\ \text{where }R^{*}=R\left(⊗E\right)+R\left(⊗H\right). \end{array}$$

#### G-TOPSIS Method

Huang and Yun proposed the TOPSIS idea in 1981, in which “*n*” alternatives are evaluated using an “*m*” number of criteria. The main goal of the TOPSIS method is to find +ve and -ve ideal solutions (variants) to a situation that have the greatest relative proximity to the pattern (+ve) and the least relative proximity to the anti-pattern (-ve). The +ve ideal solution portrays an increase in the response variable, while the -ve ideal solution portrays a decline in the response variable. Because data are not always precise in reality, the grey theory (GT) is employed to account for ambiguities. As new techniques emerge, the TOPSIS method continues to evolve. We employed this approach in conjunction with grey numbers from the GST in the current investigation. This technique is solved using the steps listed as follows (36–38).

*Stage 1*: Initially, grey numbers with the accompanying values are assigned to verbal judgments of criteria significance by the decision-makers: highly insignificant [0.0, 0.2], insignificant [0.2, 0.4], moderately significant [0.4, 0.6], significant [0.6, 0.8], and highly significant [0.8, 1.0] (39).

*Stage 2*: We use the arithmetic mean technique to aggregate the results after determining the level of significance of the decision-making criteria (*h*) by assuming the number of decision makers as *p*:


$$\begin{array}{l} ⊗w_{h}=\frac{1}{p}\left[⊗w_{h}^{1}+⊗w_{h}^{2}+\cdots +⊗w_{h}^{p}\right],\\ \text{where}:⊗w_{h}^{p}=\left[{\underline{w}}_{i^{p}},{\bar{w}}_{h}^{p}\right]. \end{array}$$

*Stage 3*: To establish the state of each of the criteria, the linguistic variables ought to be employed. The score of alternative *k* in the criteria *h* is determined by the accompanying relation, presuming that the frequency of decision makers is *p*:


$$\begin{array}{l} ⊗R_{kh}=\frac{1}{p}\left[⊗R_{kh}^{1}+⊗R_{kh}^{2}+\cdots +⊗R_{kh}^{p}\right] \end{array}$$

where $⊗R_{kh}^{p},\left(k=1,2,\cdots \;,n;h=1,2,\cdots \;,m\right)$ is an estimation of the criterion by the *p*th decision maker, which is displayed in a structure by a grey number: $⊗R_{kh}^{p}=\left[{\underline{R}}_{kh}^{p},{\bar{R}}_{kh}^{p}\right]$.

*Stage 4:* In the fourth stage, constructing the grey decision matrix in the following structure:


$$\begin{array}{l} ⊗R=\left[\begin{array}{cccc} ⊗R_{11} & ⊗R_{11} & \cdots & ⊗R_{1m}\\ ⊗R_{21} & ⊗R_{11} & \cdots & ⊗R_{2m}\\ \vdots & \vdots & \ddots & \vdots \\ ⊗R_{n1} & ⊗R_{n2} & \cdots & ⊗R_{nm} \end{array}\right]. \end{array}$$

*Stage 5*: Established the normalized grey decision matrix in the accompanying structure:


$$\begin{array}{l} ⊗R^{*}=\left[\begin{array}{cccc} ⊗R_{11}^{*} & ⊗R_{12}^{*} & \cdots & ⊗R_{1m}^{*}\\ ⊗R_{21}^{*} & ⊗R_{22}^{*} & \cdots & ⊗R_{2m}^{*}\\ \vdots & \vdots & \ddots & \vdots \\ ⊗R_{n1}^{*} & ⊗R_{n2}^{*} & \cdots & ⊗R_{nm}^{*} \end{array}\right]. \end{array}$$

If the variable attribute is beneficial, the normalization equation is as follows:


$$\begin{array}{l} ⊗R_{kh}^{*}=\left[\frac{{\underline{R}}_{kh}}{R_{h}^{max}},\frac{{\bar{R}}_{kh}}{R_{h}^{\text{max}}}\right],\text{and }R_{h}^{\text{max}}=\underset{1\leq k\leq m}{\max}\left\{{\bar{R}}_{kh}\right\}. \end{array}$$

And, on the off chance that the variable attribute is non-beneficial, the data are normalized using the accompanying equation.


$$\begin{array}{l} ⊗R_{kh}^{*}=\left[\frac{{\underline{R}}_{kh}}{R_{h}^{\text{min}}},\frac{{\bar{R}}_{kh}}{R_{h}^{\text{min}}}\right],\text{and }R_{h}^{\text{min}}=\underset{1\leq k\leq m}{\min}\left\{{\bar{R}}_{kh}\right\} \end{array}$$

The grey matrix's range will remain within [0, 1] after normalization.

*Stage 6*: Assemble the weighted normalized grey decision-making matrix in the accompanying structure:


$$\begin{array}{l} ⊗R_{\omega }^{*}=\left[\begin{array}{cccc} ⊗C_{11} & ⊗C_{12} & \cdots & ⊗C_{1m}\\ ⊗C_{21} & ⊗C_{22} & \cdots & ⊗C_{2m}\\ \vdots & \vdots & \ddots & \vdots \\ ⊗C_{n1} & ⊗C_{n2} & \cdots & ⊗C_{nm} \end{array}\right],\text{ where}C_{kh}=⊗\;R_{kh}^{*}\times ⊗\omega_{h}. \end{array}$$

*Stage 7*: Determine the ideal solution based on the assumption that given the set of “*n*” prospective alternatives *V* = {*V*_1_, *V*_2_, *V*_3_, ⋯ , *V*_*n*_}, that pattern *V*^max^ should be identified as follows:


$$\begin{array}{l} ⊗V^{max}=\left\{⊗C_{1}^{max},⊗C_{2}^{max},\cdots \;,⊗C_{m}^{\text{max}}\right\}\\ where,V^{\text{max }}=\;\{\left[\underset{1\leq k\leq n}{\max}{\underset{-}{C}}_{k1},\;\underset{1\leq k\leq n}{\max}{\bar{C}}_{k1}\right],\\ \left[\underset{1\leq k\leq n}{\max}{\underset{-}{C}}_{k2},\;\underset{1\leq k\leq n}{\max}{\bar{C}}_{k2}\right],\;\cdots \;,\;\left[\underset{1\leq k\leq n}{\max}{\underset{-}{C}}_{kn},\;\underset{1\leq k\leq n}{\max}{\bar{C}}_{kn}\right]\}. \end{array}$$

*Stage 8*: Determine the anti-ideal solution based on the assumption that given the set of “*n*” prospective alternatives *V* = {*V*_1_, *V*_2_, *V*_3_, ⋯ , *V*_*n*_}, the anti-pattern *V*^min^ should be identified as follows:


$$\begin{array}{l} ⊗V^{\text{min}}=\left\{⊗C_{1}^{\text{min}},⊗C_{2}^{\text{min}},\cdots \;,⊗C_{m}^{\text{min}}\right\}\\ where\;V^{\text{min }}=\;\{\left[\underset{1\leq k\leq n}{\min}{\underset{-}{C}}_{k1},\;\underset{1\leq k\leq n}{\min}{\bar{C}}_{k1}\right],\\ \;\left[\underset{1\leq k\leq n}{\min}{\underset{-}{C}}_{k2},\;\underset{1\leq k\leq n}{\min}{\bar{C}}_{k2}\right],\;\cdots \;,\;\left[\underset{1\leq k\leq n}{\min}{\underset{-}{C}}_{kn},\;\underset{1\leq k\leq n}{\min}{\bar{C}}_{kn}\right]\}. \end{array}$$

*Stage 9*: Estimate the distances between the alternatives under consideration, as well as the ideal (*V*^max^) and anti-ideal (*V*^min^) solutions, employing the following formulas:


$$\begin{array}{l} D_{h}^{+}=\;\overset{m}{\underset{h=1}{\sum }}D\left(C_{kh},C_{h}^{\text{max}}\right)\text{ and }D_{h}^{-}\\ =\overset{m}{\underset{h=1}{\sum }}D\;\left(C_{kh},C_{h}^{\text{min}}\right)\text{ for}h=1,2,\cdots \;,m; \end{array}$$

$\text{where}\;D\left(⊗C_{A},C_{B}\right)=\sqrt{\frac{1}{2}\left[\left(C_{A}-C_{B}\right)+\left({\bar{C}}_{A}-{\underline{C}}_{B}\right)\right]}.$

*Stage 10*: Create a synthetic assessment metric for variations *D*_*k*_ based on the relative proximity of variant evaluations to the ideal and anti-ideal solutions:


$$\begin{array}{l} D_{k}=\frac{D_{k}^{-}}{D_{k}^{+}+D_{k}^{-}}\;,k=1,2,3,\cdots \;,n. \end{array}$$

The closer the value of the measure is to 1, the minimal the interval of the assessment of the variant away from the ideal solution $\left(D_{k}^{+}\right)$, and, simultaneously, the maximum the interval away from the anti-ideal solution $\left(D_{k}^{-}\right).$

*Stage 11*: Then, in decreasing order, generate a rating for “*n*” alternatives based on linear streaming synthetic assessment metrics. The alternative with the lowest degree of grey synthetic evaluation will end up contributing more adversely to the response variable.

## Results

**Figure 1:**
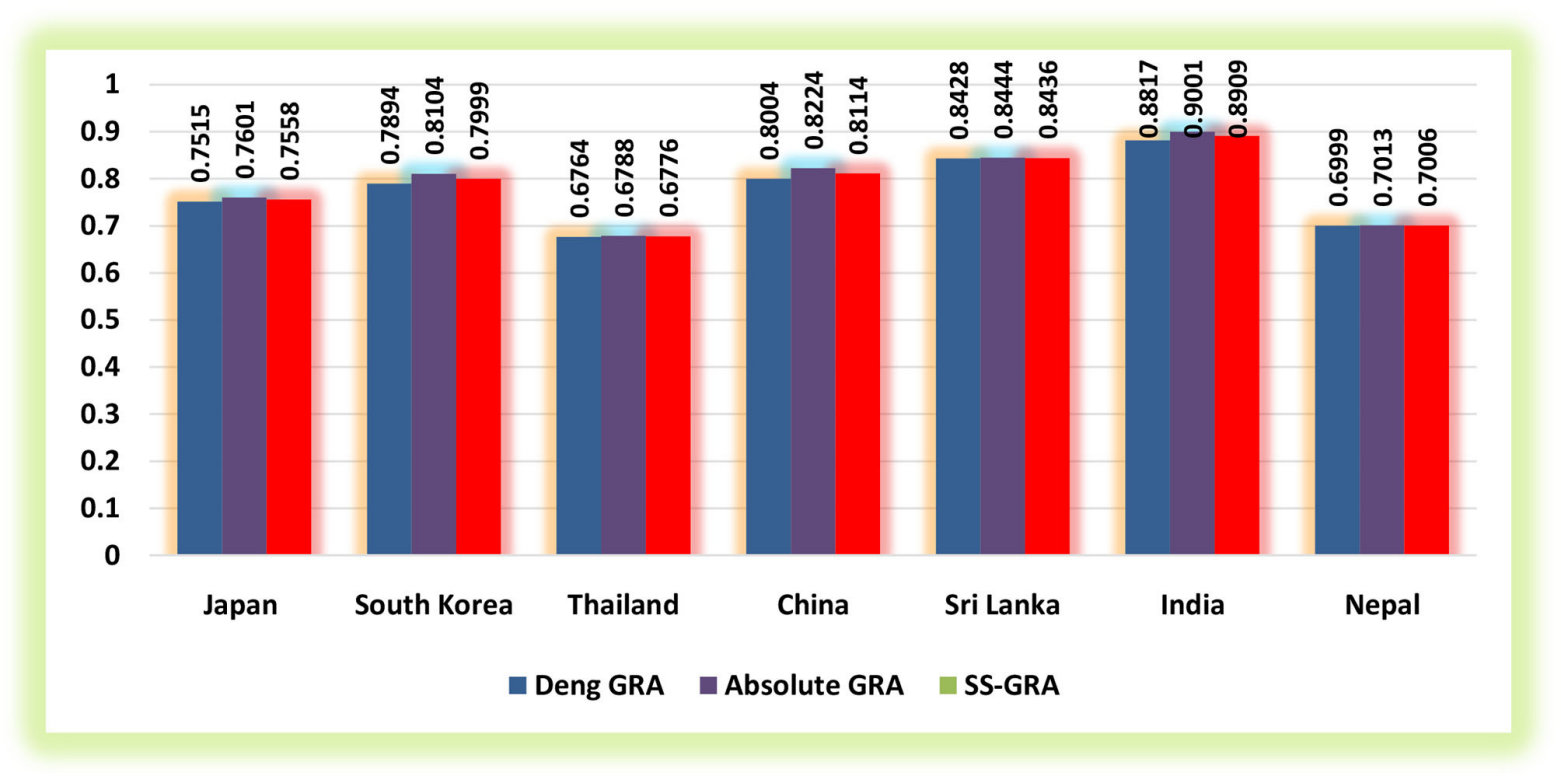
Grey relational assessment of ischemic heart disease (IHD) mortality and exposure to household PM_2.5_.

**Figure 2:**
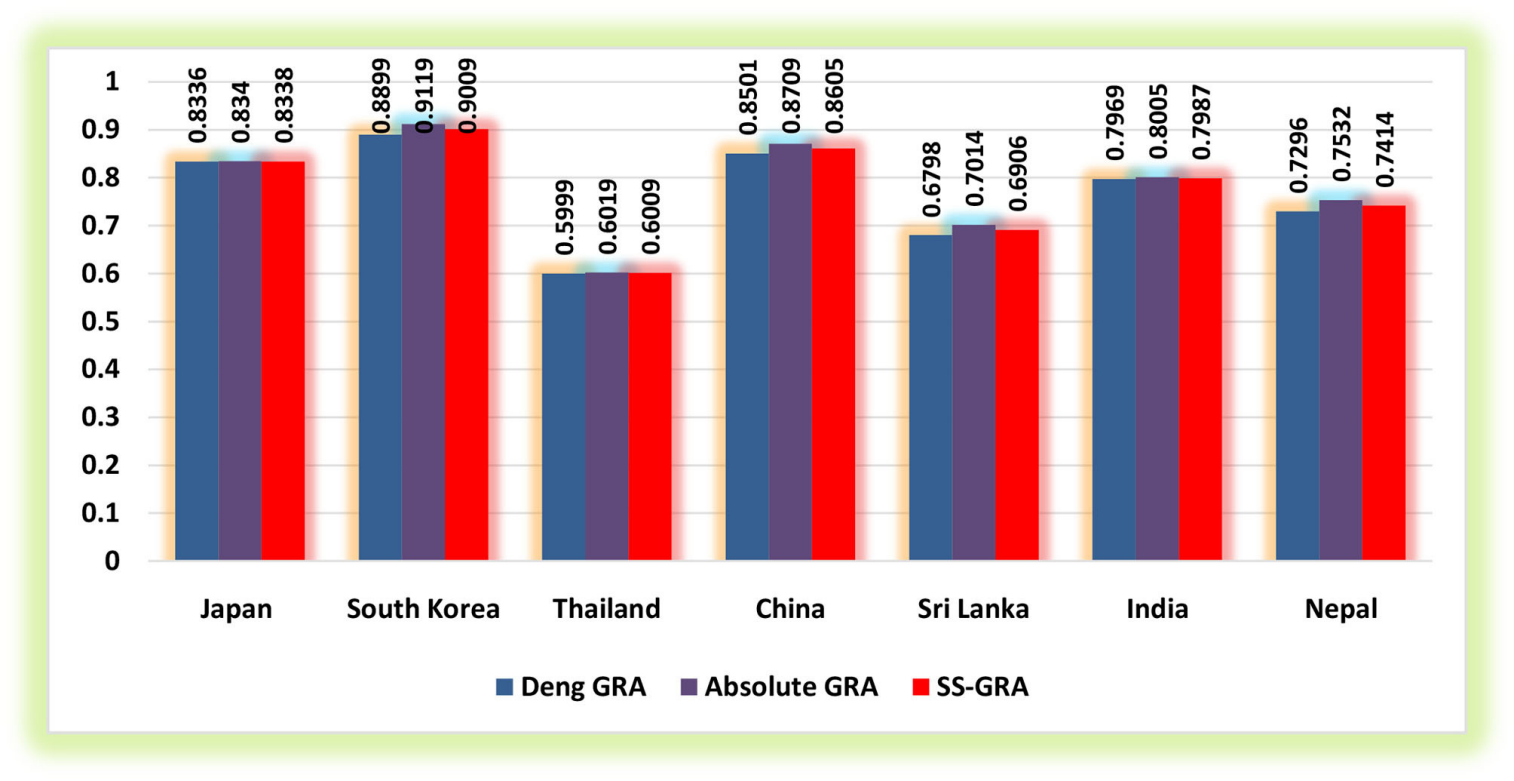
Grey relational assessment of IHD mortality and exposure to ambient PM_2.5_.

**Figure 3:**
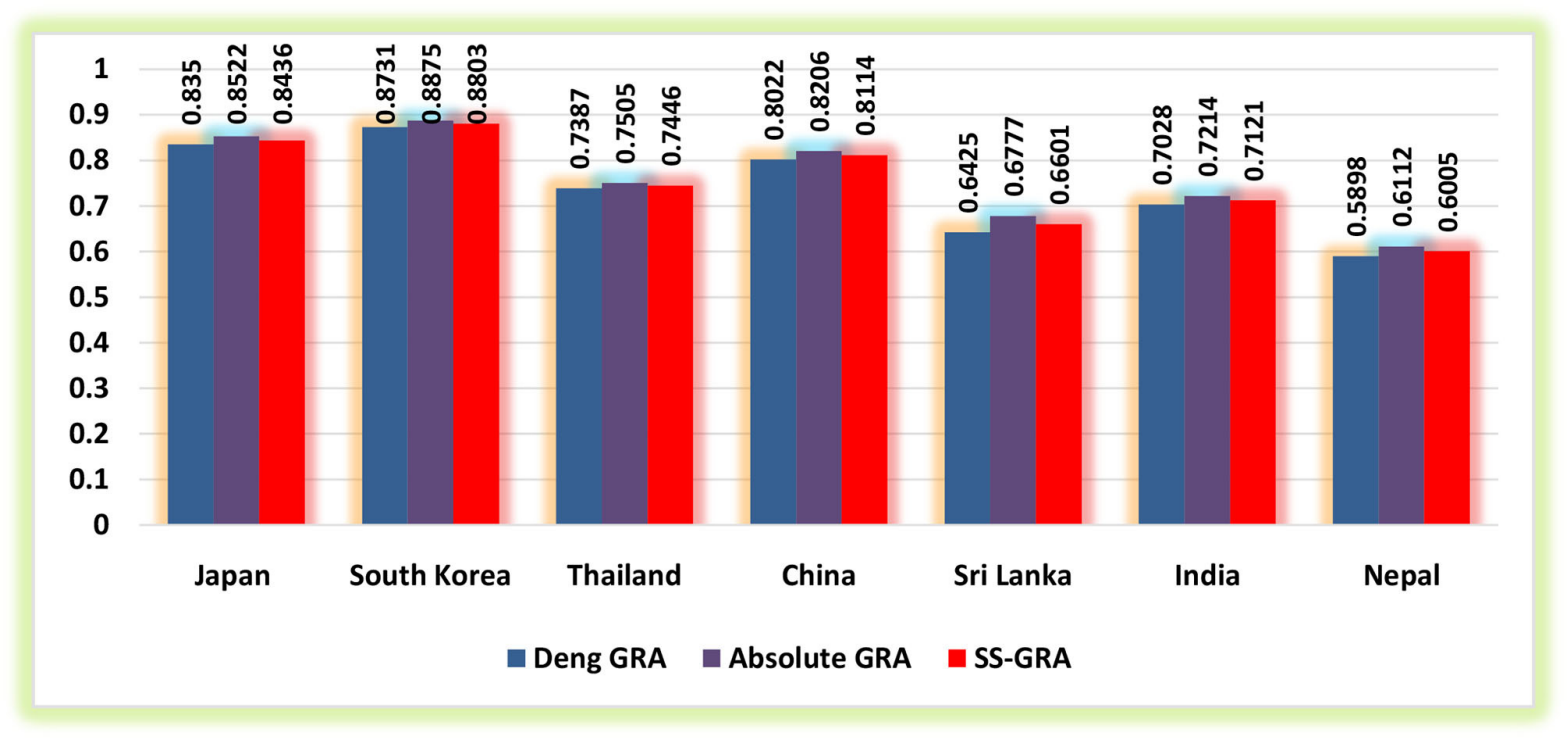
Grey relational assessment of IHD mortality and exposure to ground-level ozone (O_3_).

**Figure 4:**
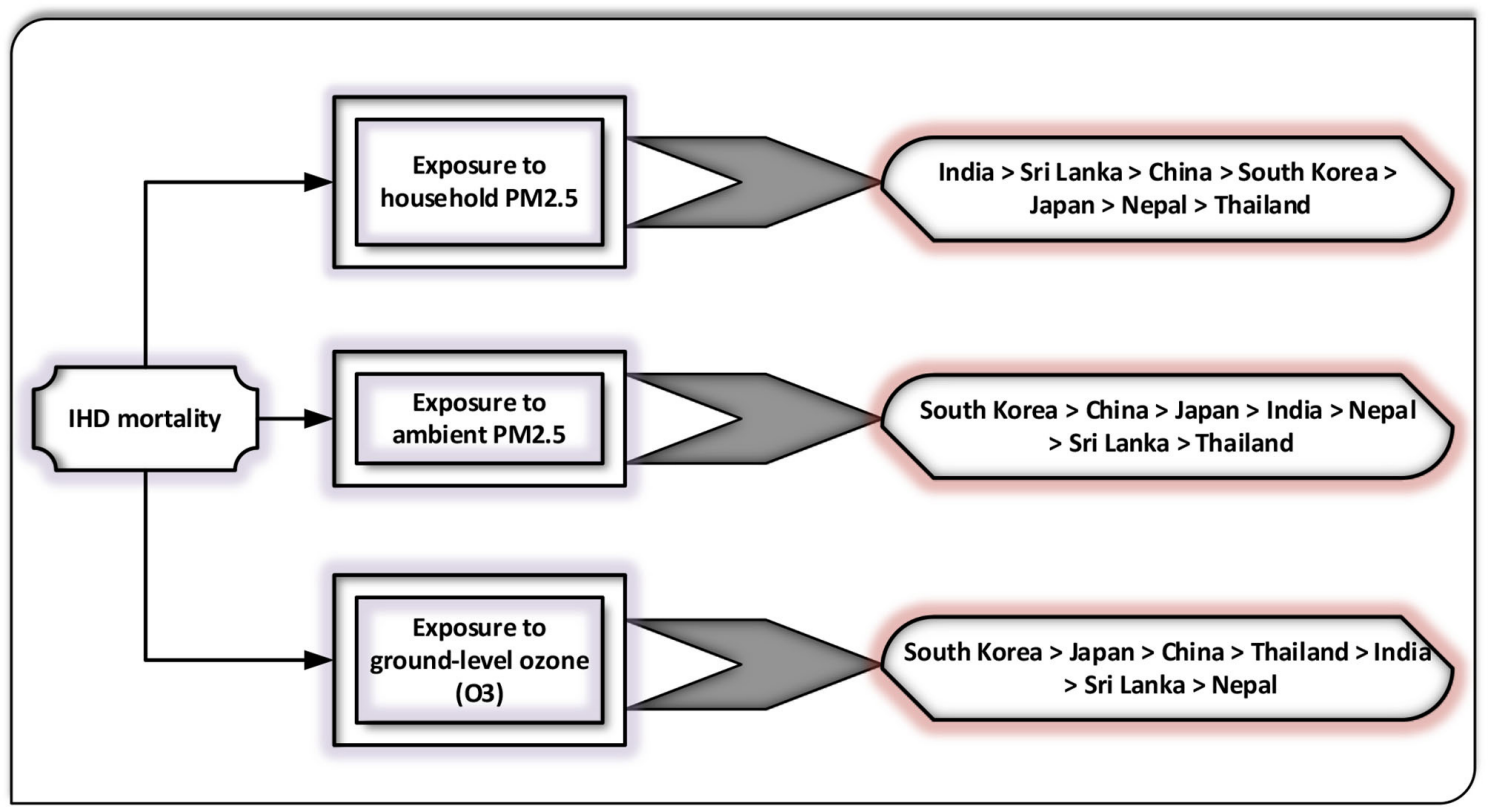
The ranking order of selected Asian countries is based on grey relational analysis (GRA).

The current study utilized the grey relational methodologies to assess the degree of proximity between exposure to household PM_2.5_, ground-level O_**3**_ exposure, exposure to ambient PM_**2.5**_, and IHD mortality for 2010–2019 in the selected regions (Japan, South Korea, Thailand, China, Sri Lanka, India, and Nepal) for the elderly populace. Tables 1–3 demonstrate the findings of grey relational models, namely, the D-GRA, A-GRA, and the SS-GRA for IHD mortality with associated factors. The A-GRA and the SS-GRA models have a scale ranging from [0, 1], while D-GRA has a scale ranging from [0.5, 1]. If the estimated value is close to 1, it is considered significantly associated and if it diverges from 1, it is considered to be weak. Table 4 shows the notations for the decision parameters. Figures 1–3 show a graphical representation of the GRA assessment between the studied variables and the air pollutants exposure. In addition, the ranking sequence based on GRA assessment can be seen in Figure 4.

**Table 1:** Grey assessment between ischemic heart disease (IHD) mortality and exposure to household PM_2.5_.

**Indicators**	**Deng GRA**	**Absolute GRA**	**SS-GRA**
Japan	0.7515	0.7601	0.7558
South Korea	0.7894	0.8104	0.7999
Thailand	0.6764	0.6788	0.6776
China	0.8004	0.8224	0.8114
Sri Lanka	0.8428	0.8444	0.8436
India	0.8817	0.9001	0.8909
Nepal	0.6999	0.7013	0.7006
Ranking sequence	India > Sri Lanka > China > South Korea > Japan > Nepal > Thailand		

**Table 2:** Grey assessment between IHD mortality and exposure to ambient PM_2.5_.

**Indicators**	**Deng GRA**	**Absolute GRA**	**SS-GRA**
Japan	0.8336	0.8340	0.8338
South Korea	0.8899	0.9119	0.9009
Thailand	0.5999	0.6019	0.6009
China	0.8501	0.8709	0.8605
Sri Lanka	0.6798	0.7014	0.6906
India	0.7969	0.8005	0.7987
Nepal	0.7296	0.7532	0.7414
Ranking sequence	South Korea > China > Japan > India > Nepal > Sri Lanka > Thailand		

**Table 3:** Grey assessment between IHD mortality and exposure to ground-level ozone (O_3_).

**Indicators**	**Deng GRA**	**Absolute GRA**	**SS-GRA**
Japan	0.8350	0.8522	0.8436
South Korea	0.8731	0.8875	0.8803
Thailand	0.7387	0.7505	0.7446
China	0.8022	0.8206	0.8114
Sri Lanka	0.6425	0.6777	0.6601
India	0.7028	0.7214	0.7121
Nepal	0.5898	0.6112	0.6005
Ranking sequence	South Korea > Japan > China > Thailand > India > Sri Lanka > Nepal		

**Table 4:** Definition of the decision parameters.

**Criteria (Countries)**	**Notations**	**Alternatives (risk factors)**	**Notations**
Japan	P_1_	Exposure to ambient PM_2.5_	RF-1
South Korea	P_2_	Exposure to ground-level ozone (O_**3**_)	RF-2
Thailand	P_3_	Exposure to household PM_2.5_	RF-3
China	P_4_		
Sri Lanka	P_5_		
India	P_6_		
Nepal	P_7_		

Table 1 summarizes the relationship between IHD mortality in the elderly and exposure to household PM_2.5_ in the selected nations using GRA models. According to D-GRA findings, the measure of influence is seen to be stronger in the case of India with an estimated weight of 0.8817 due to exposure to household PM_2.5_, followed by Sri Lanka and China. Given the determined weights for A-GRA, though the same sequence appeared to be yet the measure of association was shown up exceptionally high for India (0.9001) when compared with the rest of the countries. Notwithstanding, Thailand (0.6788) and Nepal (0.7013) are viewed as the less significant nations under grey relational assessment of exposure to household PM_2.5_ on IHD mortality among the selected regions. Overall, the strength of the relationship is significantly more grounded for India with the grey relational weight of 0.8909, suggesting that exposure to household PM_2.5_ has a considerable influence on IHD mortality in its elderly population, followed by Sri Lanka (0.8436) and China (0.8114).

Moreover, as indicated by D-GRA model findings, a more grounded measure of influence between exposure to ambient PM_2.5_ and IHD mortality in the elderly populace of South Korea (0.8899) is observed whereas the most fragile level of influence is seen for Thailand (0.5999). The higher impact measure portrays that the factors are unequivocally interconnected with one another if there should arise an occurrence of IHD mortality. Then again, the same succession showed up from the findings of the A-GRA model. The degree of correlation is found to be much higher in the case of South Korea for IHD mortality against ambient PM_2.5_ exposure with an estimated weight of 0.9119 when contrasted with the rest of the countries. At a more aggregate level, as per SS-GRA findings, exposure to ambient PM_2.5_ concentration is distinguished as a major contributor in accelerating IHD mortality in the elderly populace of South Korea. The greater degree of inclusive proximity between IHD mortality and the risk factor ambient PM_2.5_ concentrations portrays a significant association of those variables with each other (Table 2). Next to South Korea, the elderly population of China appeared to be more affected due to exposure to ambient PM_2.5_ concentrations with an estimated weight of 0.8605, and ranked second, trailed by Japan (0.8338) and India (0.7987) with comparatively less intensity.

Exposure to ground-level (surface) O_3_ concentration potentially exacerbates a multitude of health complications, including cardiovascular illnesses. Ground-level O_3_ concentrations are expected to rise in many regions of the world, resulting in an upsurge in O_3_-related deaths and morbidities (40). As per D-GRA findings, South Korea (0.8731) has appeared with the strongest influence between ground-level O_3_ exposure and mortality related to IHD, while the weakest influence is viewed for Nepal (0.5898). South Korea (0.8875) acquired the highest association led by Japan (0.8522) and China (0.8206) based on the results of A-GRA (Table 3). Again, the most fragile measure of correlation was found in Sri Lanka and Nepal, which demonstrate that these countries have the least share of disease burden due to ground-level O_3_ concentrations within the selected regions. Overall, the estimates from the SS-GRA model uncovered that among the selected economies, South Korea (0.8803) gives off an impression of being the culprit for increased IHD mortality attributable to ground-level O_3_ exposure in its elderly populace trailed by Japan and China.

### G-TOPSIS Analysis

We implemented G-TOPSIS to measure and rank the intensity of the explanatory variables (exposure to household PM_2.5_, exposure to ground-level O_**3**_, exposure to ambient PM_2.5_) on mortality from stroke and IHD for all the selected countries. We transformed the decision criteria into grey numbers through linguistic variables and then built a standardized grey decision matrix against each of the explanatory factors across all regions independently. After evaluating the weights for each criterion (countries), we then built a weighted normalized grey decision matrix (Table 5). Based on that, we determined the patterns for the ideal (RF^max^) and anti-ideal (RF^min^) solutions.

**Table 5:** Building a grey decision matrix for cardiac mortality.

	***P*_1_**	***P*_2_**	***P*_3_**	***P*_4_**	***P*_5_**	***P*_6_**	***P*_7_**
**IHD mortality**
**Normalized grey decision matrix**
RF-1	[0.45,0.83]	[0.26,0.90]	[0.40,1.00]	[0.64,0.96]	[0.44,0.88]	[0.47,1.00]	[0.42,0.92]
RF-2	[0.30,0.75]	[0.25,0.75]	[0.62,1.00]	[0.59,1.00]	[0.48,1.00]	[0.55,0.88]	[0.44,0.82]
RF-3	[0.37,1.00]	[0.44,0.85]	[0.50,0.88]	[0.53,0.78]	[0.29,0.65]	[0.36,0.77]	[0.76,1.00]
**Weighted normalized grey decision matrix**
RF-1	[0.28,0.66]	[0.15,0.65]	[0.28,0.88]	[0.44,0.79]	[0.30,0.77]	[0.33,0.88]	[0.31,0.74]
RF-2	[0.19,0.60]	[0.15,0.54]	[0.43,0.88]	[0.40,0.82]	[0.33,0.88]	[0.39,0.77]	[0.33,0.66]
RF-3	[0.23,0.80]	[0.26,0.61]	[0.35,0.77]	[0.36,0.64]	[0.20,0.57]	[0.25,0.68]	[0.56,0.80]
**Ideal and anti-ideal patterns**
RF_max_	[0.28,0.80]	[0.26,0.65]	[0.43,0.88]	[0.44,0.82]	[0.33,0.88]	[0.39,0.88]	[0.56,0.80]
RF_min_	[0.19,0.60]	[0.15,0.54]	[0.28,0.77]	[0.36,0.64]	[0.20,0.57]	[0.25,0.68]	[0.31,0.66]

Using the findings in Table 5, we computed distances of the alternatives (RF-1, RF-2, and RF-3) from ideal and anti-ideal patterns against each criterion (countries), which are presented in Table 6.

**Table 6:** Estimated distances of the alternatives (RF_*h*_) from the ideal and anti-ideal pattern.

***D* ^+^**							
RF-1	0.07	0.06	0.08	0.02	0.07	0.03	0.16
RF-2	0.15	0.11	0.00	0.02	0.00	0.06	0.19
RF-3	0.03	0.02	0.10	0.13	0.22	0.17	0.00
***D* ^−^**							
RF-1	0.08	0.06	0.06	0.12	0.15	0.14	0.04
RF-2	0.00	0.00	0.13	0.11	0.22	0.12	0.01
RF-3	0.12	0.09	0.04	0.00	0.00	0.00	0.20

Based upon the calculated distances (Table 6), we established grey synthetic assessment measures of the risk factors (RF-1, RF-2, and RF-3) for mortality from stroke and IHD (Table 7) and then created a ranking pattern in a descending order, which can be seen in Table 7.

**Table 7:** Estimated grey degree of synthetic assessment.

**Parameters**	$\sum D_{k}^{+}$	$\sum D_{k}^{-}$	$\sum D_{k}^{+}+\sum D_{k}^{-}$	$D(A_{h})=\frac{\sum D_{k}^{-}}{\sum D_{k}^{+}+\sum D_{k}^{-}}$	**Optimal solution**
IHD
RF-1	0.49	0.65	1.14	0.57	Max
RF-2	0.53	0.59	1.12	0.52	
RF-3	0.67	0.45	1.12	0.40	Min
Ranking	Exposure to ambient PM_**2.5**_ **>** Exposure to ground-level ozone (O_**3**_) > Exposure to household PM_2.5_				

**Figure 5:**
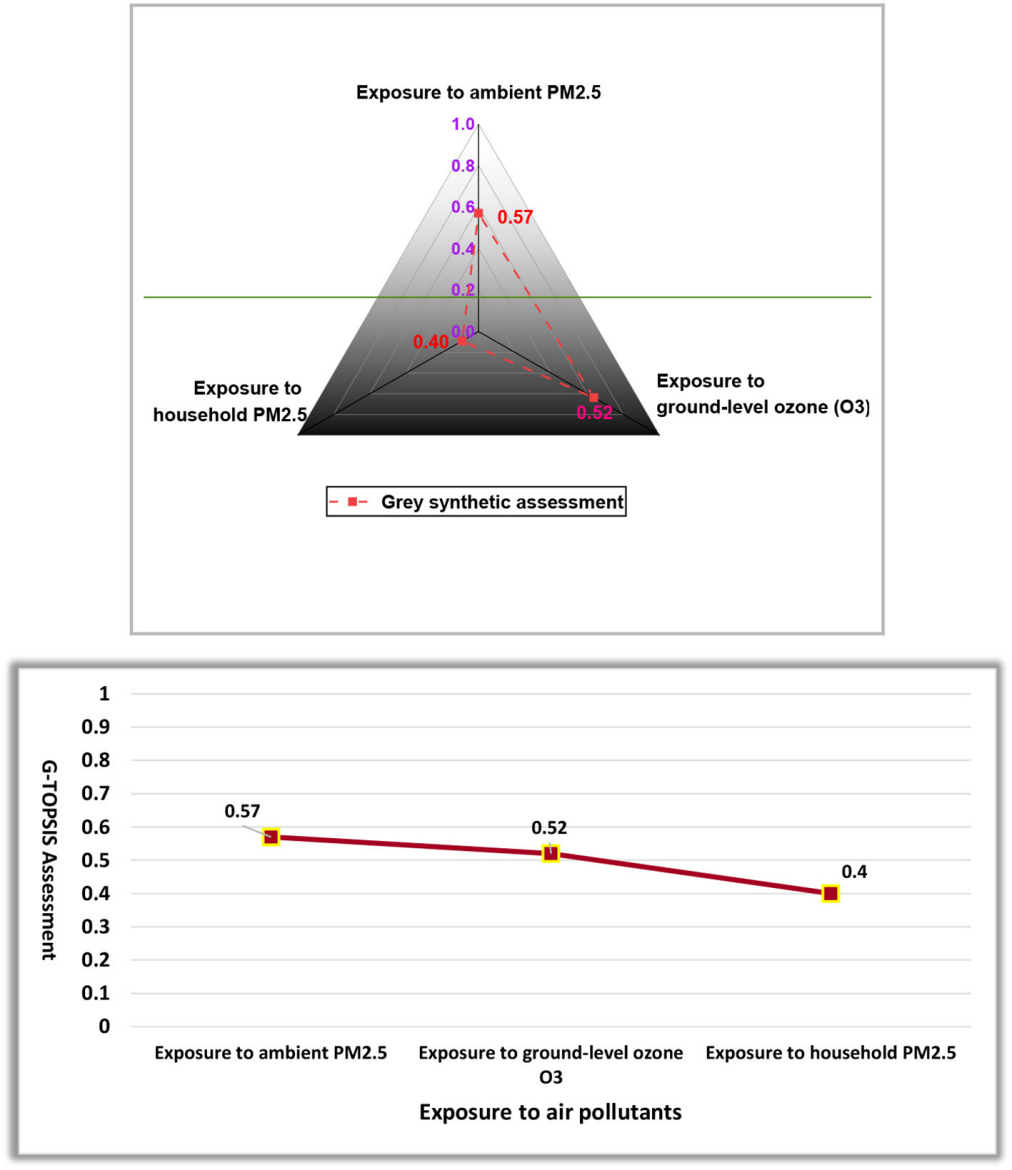
Grey-based Technique for Order of Preference by Similarity to Ideal Solution (G-TOPSIS) assessment of IHD-related risk factors.

Based on the outcomes of the G-TOPSIS synthetic assessment, an interesting ranking sequence appeared for IHD mortality. Therefore, it is concluded that exposure to household PM_2.5_ (0.40) gives off an impression of being the worst factor in escalating mortality associated with IHD trailed by ground-level O_3_ exposure (0.52) and ambient PM_2.5_ exposure (0.57). The use of multi-criteria decision analysis (MCDA) approaches in the current study will support public health policymakers in making decisions on the best strategy to reduce cardiac mortality in the targeted regions by focusing on the worst factors. The graphical representation of grey based TOPSIS assessment can be seen in Figure 5.

## Discussion and Conclusions

To confront the environmental impact on public health, the present study takes a step forward in investigating the degree of proximity between exposure to ambient PM_2.5_, household PM_2.5_, ground-level O_**3**_, and IHD mortality among the top seven Asian countries with the highest rate of the elderly population, employing the time-series data for the period 2010–2019. This research has been carried out using three grey relation models D-GRA, A-GRA, and SS-GRA models, which may be implemented as a viable alternative to conventional data analysis approaches. According to GRA findings, South Korea appeared to be one of the most affected Asian regions due to exposure to ambient PM_2.5_ and ground-level O_3_ in terms of IHD mortality. In contrast, India showed up as the biggest contributor of IHD mortality among the studied economies owing to household PM_2.5_ exposure. In addition, the findings of G-TOPSIS highlighted that, among the chosen parameters (risk factors), exposure to household PM_2.5_ concentrations is the most potential risk factor in raising IHD mortality across the selected Asian economies.

Given the rapid growth in the aging population of South Korea, the morbidity and mortality associated with IHD in the elderly are escalating (41). In our analysis, exposure to ambient PM_2.5_ and ground-level O_3_ appeared as a potential contributor to IHD mortality in the population of South Korea when compared with the rest of the selected Asian nations. The outcomes of our study are both consistent and conflicting with previous epidemiological studies, which investigated these associations using traditional statistical approaches, but our study differs from a methodological standpoint in that we evaluated the degree of proximity among the selected variables while keeping endogeneity issues in mind. The assessment of the degree of proximity is higher for the elderly in South Korea. Particulate air pollutants (PM2.5, PM1, or PM10) provide the most compelling evidence for the influence of air pollutants on mortality. In a study conducted in Singapore, PM_2.5_ had a substantial influence on cardiac mortality in the elderly but not in the non-elderly when compared to other air pollutants such as carbon monoxide (CO), nitrogen dioxide (NO_**2**_), and surface O_**3**_ (42). Significant correlations between particulate matter exposure and different short- and long-term cardiac health outcomes for the elderly were revealed in the review research conducted between 1991 and 2016. The American Heart Association (AHA) emphasized the clinical relevance for academics and healthcare practitioners in a much more extensive analysis of the recent findings associated with particulate matter exposure to CVD. Long-term exposure to PM_2.5_ has been demonstrated in studies to induce IHD-related mortality and nonfatal incidents and may exacerbate the detrimental effects on microvascular functioning and an increased risk of IHD mortality (43, 44). Reduced particulate matter concentrations, however, are related to reductions in IHD mortality in as little as a few years. In this view, the improvement in quality of life and related health outcomes owing to lower concentrations of air pollutants might be seen by the population in a few years, essentially leading to the adoption of more rigorous policy choices on air quality in most parts of the world (9, 13, 45).

Few studies have been undertaken to assess the impact of ground-level O_3_ on population-based comparative research in the elderly in the selected Asian countries. Exposure to another pollutant, which is surface O_3_ a constituent of the photochemical air pollution combination, might exacerbate the negative health impacts of particulate matter. Some epidemiologic studies have confirmed that O_3_ exposure has a major impact on human health (42, 46, 47). A few of the health impacts of O_3_ include vascular system inflammation, a variation in heart rates, and a drop in the capacity of blood clots to disintegrate, all of which are the risk factors for heart disease (48). All of these consequences can increase vulnerability to infections and, in the end, result in a cardiac catastrophe. According to a European study, an elevation of 10 g/m^3^ within 1–8 h surface O_3_ level exposure increases the risk of mortality by 1.13 and 0.33% on the overall daily number of fatalities related to respiratory and cardiac deaths, respectively (49). In this study, a higher degree of proximity is observed in the population of South Korea, implying the need for improved public policies to address air quality in this region. In South Korea, the two major causes of air pollution are emissions from fossil fuel combustion and vehicular emissions (50). South Korea's economy grew at a 10% annual rate in the 1980s and 1990s. In 2015, South Korea had been the world's 11th highest gross domestic producer; however, this was achieved through polluting coal-fired power plants and dirty vehicular emissions (51, 52). To persuade individuals to leave their automobiles at home and travel by public transportation, an effective public transportation framework is essential. Clean, renewable energy and power generation can be used to operate modern buses. Many trains are also powered by electricity. When utilized within borders, delivery vehicles may be limited to using exclusively electric power. Electricity generation does not have to rely solely on fossil fuels. Cleaner technology, such as renewable energy, combined with energy storage and greater energy efficiency, can contribute toward a more sustainable energy infrastructure with a minimal environmental impact (53, 54).

According to the GRA, household exposure of PM_2.5_ appeared to be an intensified risk factor in the dense population of India with a greater degree of proximity. In addition, the G-TOPSIS outcomes also showed exposure to household PM_2.5_ as the potential risk factor for IHD-related mortality among the selected Asian regions. In India, industries have grown at an exponential rate; thusly, urbanization degrades environmental quality indirectly *via* industrialization (55, 56). Furthermore, due to the poor public transit infrastructure in India's urban regions, inhabitants opt for private transportation, which has led to massive automobile emissions that contribute to environmental deterioration. Consequently, urbanization in India causes environmental pollution and worsens population health over the long haul (57). As per the IQAir report, India's air quality is unhealthy; the most current statistics show that the country's annual mean concentration exceeds the recommended level of 10 g/m^3^ (58). The most significant contributors to air pollution in metropolitan areas have been inadequate energy consumption, a spike in the number of vehicles driven regularly, an increase in uncontrolled industrial emissions, and the combustion of waste and plastic. Thusly, for a synergistic reduction of air pollution, a holistic management framework integrating health, energy, climate, and environment sectors should be designed to mitigate IHD mortality.

Individually, the amount of fuel burned in a household might be significantly less than the amount used in industries. However, its influence on population health is far stronger because of its pervasive and continuous existence in the internal environment and the maximum time spent inside humans. This issue is quite possibly the most ignored area of the disease burden in these nations. It is indeed not hard to establish a tight connection between smoke (biomass fuel combustion) exposure and health risks in humans. To minimize household PM_2.5_ concentrations during culinary activities, a variety of treatments are available. Changes in energy technology and boosting public awareness about the severity of household PM_2.5_ concentrations caused by cooking are required at regional levels. Appropriate measures tending to a wide variety of issues related to cooking through awareness, economic development, and renewable energy resources can be extremely beneficial in reducing the possible cardiac health concerns produced by biomass fuel smoke.

In conclusion, these findings have considerable implications for public health strategy and decision makers in perspectives of the sustainable development goals (SDGs) of good health and a sustainable environment. We must acknowledge that the health industry is only one of the many aspects to attain a health goal with a CVD focus. Environmental sustainability will be influenced by agricultural, environmental, public transit, and economic policy changes as well as international trade agreements. We should cooperate and work in collaboration across regions and disciplines to advance and insinuate a profitable return of interest in heart wellbeing; only this way we could convince economies and businesses to make contributions to our mutual objectives, which are critical to the global population health and wellbeing. Ultimately, Asian governments should work collaboratively, empower and focus on the strategies that can mitigate the growing burden of CVD *via* planned urbanization and industrialization, acceptance of clean and renewable energy resources, increased educational attainment, improved and better living standards, managed to improve access to healthcare services, and spending on public health to reduce the risk of air pollutants and their associated CV mortality. We believe that raising knowledge about CV risk factors, prevention, treatment, and care in the Asian region would need a multi-sectoral partnership including all stakeholders.

## Data Availability Statement

The data used to support the findings of this study are included within the article.

## Ethics Statement

Ethical review and approval was not required for the study on human participants in accordance with the local legislation and institutional requirements. Written informed consent for participation was not required for this study in accordance with the national legislation and the institutional requirements.

## Author Contributions

AM, SR, NR, and AH are responsible for conceptualizing the research theme, data collection and analysis, interpretation of the results, and drafting earlier versions of the manuscript. SR was in charge of project administration and supervision of the overall manuscript. All authors read and approved the final version.

## Conflict of Interest

The authors declare that the research was conducted in the absence of any commercial or financial relationships that could be construed as a potential conflict of interest.

## Publisher's Note

All claims expressed in this article are solely those of the authors and do not necessarily represent those of their affiliated organizations, or those of the publisher, the editors and the reviewers. Any product that may be evaluated in this article, or claim that may be made by its manufacturer, is not guaranteed or endorsed by the publisher.
